# Antiviral Role of IFITM Proteins in African Swine Fever Virus Infection

**DOI:** 10.1371/journal.pone.0154366

**Published:** 2016-04-26

**Authors:** Raquel Muñoz-Moreno, Miguel Ángel Cuesta-Geijo, Carles Martínez-Romero, Lucía Barrado-Gil, Inmaculada Galindo, Adolfo García-Sastre, Covadonga Alonso

**Affiliations:** 1 Department of Biotechnology, Instituto Nacional de Investigación y Tecnología Agraria y Alimentaria (INIA), Madrid, Spain; 2 Department of Microbiology, Icahn School of Medicine at Mount Sinai, New York, United States of America; 3 Global Health and Emerging Pathogens Institute, Icahn School of Medicine at Mount Sinai, New York, United States of America; 4 Department of Medicine, Division of Infectious Diseases, Icahn School of Medicine at Mount Sinai, New York, United States of America; Friedrich-Loeffler-Institut, GERMANY

## Abstract

The interferon-induced transmembrane (IFITM) protein family is a group of antiviral restriction factors that impair flexibility and inhibit membrane fusion at the plasma or the endosomal membrane, restricting viral progression at entry. While IFITMs are widely known to inhibit several single-stranded RNA viruses, there are limited reports available regarding their effect in double-stranded DNA viruses. In this work, we have analyzed a possible antiviral function of IFITMs against a double stranded DNA virus, the African swine fever virus (ASFV). Infection with cell-adapted ASFV isolate Ba71V is IFN sensitive and it induces IFITMs expression. Interestingly, high levels of IFITMs caused a collapse of the endosomal pathway to the perinuclear area. Given that ASFV entry is strongly dependent on endocytosis, we investigated whether IFITM expression could impair viral infection. Expression of IFITM1, 2 and 3 reduced virus infectivity in Vero cells, with IFITM2 and IFITM3 having an impact on viral entry/uncoating. The role of IFITM2 in the inhibition of ASFV in Vero cells could be related to impaired endocytosis-mediated viral entry and alterations in the cholesterol efflux, suggesting that IFITM2 is acting at the late endosome, preventing the decapsidation stage of ASFV.

## Introduction

Upon infection with pathogens such as bacteria or viruses, the host cell activates the innate immune response as a first line of defense. The group of cytokines known as interferons (IFN) plays a major role in the cell immunity by inducing a cascade of interferon-stimulated genes (ISGs) that encode for several antiviral innate immune effectors. Among ISGs, the interferon-induced transmembrane proteins (IFITMs) are known to inhibit entry of a wide variety of enveloped RNA viruses [1]. This group of proteins is present across a wide range of species: from amphibians, fish and birds to mammals. IFITMs in humans were identified 26 years ago as interferon-stimulated genes upon induction of type-I and type-II IFN [2, 3]. Human IFITM1, IFITM2 and IFITM3 are expressed in almost every cellular type, whereas IFITM5 is expressed primarily in osteoblasts, as it is required for bone mineralization [4].

IFITMs are found mainly distributed at the plasma membrane and/or at endosomal membranes. The IFITM1, 2, 3 and 5 genes are clustered on chromosome 11, and they encode for relatively small proteins (about 130 amino acids) with both extra-cytoplasmic termini separated by two transmembrane domains (TM1 and TM2) and a cytoplasmic loop (CIL) [3] [5]. TM1 and the CIL are well conserved between the IFITM proteins and a large group of members of the CD225 protein family.

IFITM 1, 2, and 3 are currently known to inhibit the replication of multiple RNA viruses that enter the host cell via endocytosis, including influenza A virus (IAV), West Nile virus (WNV), Dengue virus (DENV) [6], severe acute respiratory syndrome coronavirus (SARS CoV) and hepatitis C virus (HCV) [7]. In contrast, IFITMs do not inhibit the entry process of mouse leukemia virus (MLV), Machupo virus (MACH), Lassa virus (LASV) or lymphocytic choriomeningitis virus (LCMV) [4].

Little is known about the IFITM-mediated antiviral activity against DNA viruses. Only IFITM1 has been recently described to inhibit Rana grylio virus (RGV), a frog/fish iridovirus, at the entry stage [8]. On the other hand, IFITM1, 2 and 3 have been reported not to affect the replication of other DNA viruses, such as human papillomavirus (HPV), human cytomegalovirus (HCMV) and adenovirus 5 (Ad5) [9].

The antiviral effect of IFITMs is mainly exerted through their effects on the endocytic pathway and would affect viruses entering the cell through a late endosomal compartment [4]. To further expand our understanding on the antiviral activity of IFITMs against DNA viruses, we investigated the role of these proteins in the replication cycle of the African swine fever virus (ASFV), belonging to the nucleocytoplasmic large DNA virus (NCLDV) superfamily [10]. ASFV infection is strongly dependent on the endocytic pathway [11, 12], thus a possible IFITM-mediated inhibition of the virus could likely occur in the endosomal compartments.

ASFV is the only member of the *Asfarviridae* family and is responsible of a highly lethal and hemorrhagic disease affecting domestic swine, which often results in important economic losses in many countries due to the high rate of mortality associated with the illness and the lack of an effective vaccine [13]. An epidemic outbreak is currently affecting East Europe and is slowly spreading between neighboring countries [14–16]. We previously reported that ASFV enters into the host cell by dynamin-dependent and clathrin-mediated endocytosis [12, 17]. Thus, our goal in the current work was to test whether the IFITM family of proteins affected early entry steps of ASFV infection in Vero cell cultures using the cell-adapted Ba71V isolate.

## Materials and Methods

### Cell culture and viruses

Vero cells were obtained from the American Type Culture Collection (ATCC, Richmond, VA, USA) and maintained in Dulbecco modified Eagle medium supplemented with 5% fetal bovine serum (FBS), 100 lU/ml penicillin, 100ug/ml streptomycin and 2mM L-glutamine at 37°C at 5% CO_2_. Cells were pretreated with 1,000 or 10,000 U/ml of universal type-I IFN (PBL Assay Science) for 24 h, as indicated.

The tissue culture-adapted ASFV isolate Ba71V was used in most experiments [18]. For flow cytometry analyses, a recombinant virus expressing the viral protein p54 fused to the green fluorescent protein (GFP) was used (B54GFP) [19]. Preparation of viral stocks, titrations and infection procedures were carried out in Vero cells as previously described [18]. When synchronization of viral infection was required, the adsorption phase took place at 4°C to allow viral attachment to the cell surface but impeding its internalization. When indicated, ASFV was semi-purified by sucrose cushion (40%) in PBS at 40,000xg for 50 min at 4°C.

### Generation of stable cell lines

To generate stable cell lines expressing different proteins, the commercially available lentiviral expression vector pLVX-Puro (Clontech) was used to clone the proteins of interest. 293T cells were transfected at 100% confluency using Lipofectamine 2000 (Life technologies) with Opti-MEM (Life technologies) in 10-cm^2^ plates. Plates were previously pretreated with poly-L-lysine (Sigma-Aldrich) at a final concentration of 0.1 mg/ml to avoid cell detachment. Co-transfection of pLVX-puro expression vector together with vesicular stomatitis virus glycoprotein (VSV-G) and the human immunodeficiency virus (HIV) gag-pol expressing plasmids was performed to produce pseudotyped lentiviral vectors.

Supernatants containing the pseudotyped lentiviruses were collected twice at 48 h and 72 h postransfection. Cell debris was removed by brief centrifugation at 1,000 rpm for 5 min and cleared supernatants were 0.22 μM-filtered and stored at -80°C until use.

Sub-confluent Vero cells were transduced with the pseudotyped lentiviruses expressing the gene of interest and supplemented with 1 μg/ml of polybrene (EMD Millipore). 24 h later, transduced cells were selected by adding 8 μg/ml of puromycin (Life Technologies). Optimal puromycin working concentration was previously titrated in non-transduced cells. Finally, protein expression levels of Vero stable-cell lines were determined by Western Blot (WB).

### Immunofluorescence

Cells were seeded and grown on glass coverslips. Mock-infected and infected cells were fixed with 4% paraformaldehyde in PBS for 15 min at room temperature (RT). Following cell fixation, aldehyde fluorescence was quenched by incubation of cells with 50 mM NH_4_Cl in PBS for 10 min. Then, cells were permeabilized with PBS–0.1% Triton X-100 or Saponin (Sigma) for 10 min at RT.

After blocking with bovine serum albumin (BSA; Sigma) or normal goat serum (Sigma), cells were stained with primary and secondary antibodies and then incubated with Topro-3 (Molecular Probes) in PBS at a 1:1,000 ratio for DNA staining. After washing, coverslips were finally mounted on glass plates and cells were observed under a Leica TCS SP2-AOBS confocal microscope (Leica-Microsystems, Wetzlar, Germany) using a 63X immersion oil objective.

To detect cholesterol distribution we used Filipin staining (Sigma), as previously described [20]. Cholesterol was visualized in a conventional Leica DM RB microscope by combining a 63X immersion oil objective and a UV filter set. Images were captured with Leica Application Suite advanced fluorescence software (LAS AF) and ImageJ software. Finally, digital images were processed with Adobe Photoshop 8.0.

The primary antibodies used for immunofluorescence assays included the following: rabbit polyclonal antibodies to IFITM1, IFITM2 and IFITM3 (Proteintech), 1:200; anti-ASFV p30 mouse monoclonal antibody, 1:100 (kindly given by Dr. J.M. Escribano, INIA); ASFV mouse monoclonal antibodies anti-p72 (clone 1BC11 for immunofluorescence 1:1,000 or clone 18BG3 for WB 1:2,000) and anti-p150 (clone 17AH2, Ingenasa), 1:1,000; mouse monoclonal to CD63 (Developmental Studies Hybridoma Bank, University of Iowa, clone H5C6), 1:200; rabbit polyclonal to Lamp1 (Abcam), 1:50; rabbit polyclonal to EEA1 and Rab7 (Cell Signalling), 1:50.

Secondary antibodies were purchased from Molecular Probes and diluted 1:200. Specificity of labeling and absence of signal crossover were determined by examination of single labeled control samples.

### Western Blotting

Cells were harvested in Sample Buffer Laemmli 2X concentrate (Sigma). Then, the samples were incubated for 5 min at 95°C and resolved by SDS-PAGE in 12% or 7% polyacrylamide-bisacrylamide gels. Afterwards, separated proteins were transferred to a nitrocellulose membrane (Bio-Rad) and the non-specific antibody binding sites were blocked with skimmed milk diluted in PBS and then incubated with the specific primary and HRP (Horseradish peroxidase)-conjugated secondary antibodies. Antibodies used for western blotting included: rabbit polyclonal to IFITM1, IFITM2 and IFITM3 (Proteintech), 1:2,000; anti-ASFV p30 mouse monoclonal antibody, 1:500; anti-ASFV p72 mouse monoclonal antibody (clone 1BC11, Ingenasa), 1:1,000, and mouse monoclonal to tubulin (Sigma-Aldrich), 1:2,000. As secondary antibody, anti-mouse IgG (GE Healthcare) or anti-rabbit IgG (Bio-Rad) conjugated to horseradish peroxidase was used at a 1:5,000 dilution. Precision Protein StrepTactin-HRP Conjugate (Bio-Rad) was used to reveal the ladder Precision Plus Protein WesternC (Bio-Rad). As a loading control an anti-mouse antibody against β-tubulin (Sigma) was used, 1:2,000. Finally, bands obtained after development with ECL reagent (GE Healthcare, Piscataway, NJ, USA) were detected using a Chemidoc XRSplus Imaging System (Bio-Rad). Band densitometry was performed with Image Lab software (Bio-Rad) and normalized to control values.

### Detection and quantitation of the ASFV genome

The quantitation of the number of copies of ASFV genome was achieved by quantitative real-time PCR (qPCR) using specific oligonucleotides and a Premix ExTaq (TM) (2X; Takara) probe. Fluorescent hybridization probes were used to amplify a region of the p72 viral gene, as described previously [21]. DNA from cells mock-infected or infected with ASFV Ba71V at MOI of 1 pfu/cell was extracted at 16 hpi and purified with a DNAeasy blood and tissue kit (Qiagen). DNA concentration was measured using Nanodrop. The amplification mixture was prepared on ice as follows: 250 ng template DNA diluted in mQ H_2_O to a total volume of 7μl, 1 μl oligonucleotide OE3F (50 pmol), 1 μl oligonucleotide OE4R (50 pmol), 10 μl PCR Premix Ex Taq(TM) (2X; Takara), 1 μl TaqMan probe SE2 (5 pmol) [21]. Positive amplification controls included DNA purified from ASFV purified virions at different concentrations as standards. Negative amplification controls consisted in DNA from mock-infected cells. Each sample was included in triplicates and values were normalized to standard positive controls. Reactions were performed using the ABI 7500 Fast Real-Time PCR System (Applied Biosystems) with the following parameters: 1 cycle at 94°C for 10 min, 45 cycles at 94°C for 15 s, and 45 cycles at 58°C for 1 min.

### Decapsidation assay

To study virion decapsidation in ASFV, a protocol was adapted from a previous publication [22]. Briefly, stable cell lines Vero-IFITM1, IFITM2, IFITM3 or control cells containing the empty vector were infected at MOI of 10 pfu/cell after viral synchronization at 4°C for 90 minutes to enable virus attachment to the cell but restricting viral entry. Infection was allowed to proceed for 75 minutes at 37°C, 5% CO_2_. Cells were then washed with cold PBS 1X and treated with 0.05% trypsin-EDTA (Gibco) for 10 minutes at 37°C to remove the membrane-bound virus. Finally, cells were placed in media containing FCS to quench trypsin activity and washed with PBS. After this treatment, only internalized virions were observed in an immunofluorescence assay as described above. We used specific antibodies to detect the major viral capsid protein p72 and the viral core protein p150, and staining was analyzed by confocal microscopy. Decapsidated virions were single labeled for p150 and were counted for each condition and normalized to the total number of fully encapsidated virions which were double labeled for p72 and p150.

### Flow cytometry

Stable cell lines Vero-IFITM1, IFITM2, IFITM3 or control cells containing the empty vector were infected with Ba71V or B54GFP at the indicated MOI. Recombinant B54GFP is a recombinant ASFV expressing green fluorescent protein as a fusion protein of viral p54 [19]. Samples infected with B54GFP at 16 hpi were just fixed and washed with FACs buffer three times before analysis.

Vero cells infected with Ba71V at 6 hpi or 16 hpi (early or late postinfection times respectively), were harvested by trypsinization, washed with FACS buffer (containing PBS, 0,01% sodium azide, and 0,1% bovine serum albumin), fixed and permeabilized with Perm2 (BD science) for 10 min at RT and finally incubated with specific primary antibodies against p30 and p72 for 30 min at 4°C. The secondary antibody used was phycoerythrin (PE) conjugated (DAKO) 1:50 diluted in FACS buffer for 30 min at 4°C. After repeated washes, 10,000 cells/tube were analyzed in the FACSCalibur flow cytometer (BD Science) in triplicates. The obtained infection rates were always normalized to the corresponding control.

### Statistical analysis

Bonferroni’s multiple-comparison test was used to compare different experimental groups. Prism software (GraphPad Software, Inc.) and INSTAT3 software were used for the statistical analysis. Values were expressed in graph bars as mean ±SD of at least three independent experiments unless otherwise noted. Metrics were normalized to control values and represented in graphics. Asterisks denote statistically significant differences (***p<0.001, **p<0.01 and *p<0.05).

## Results

### IFN treatment abrogates ASFV infection

**Fig 1:**
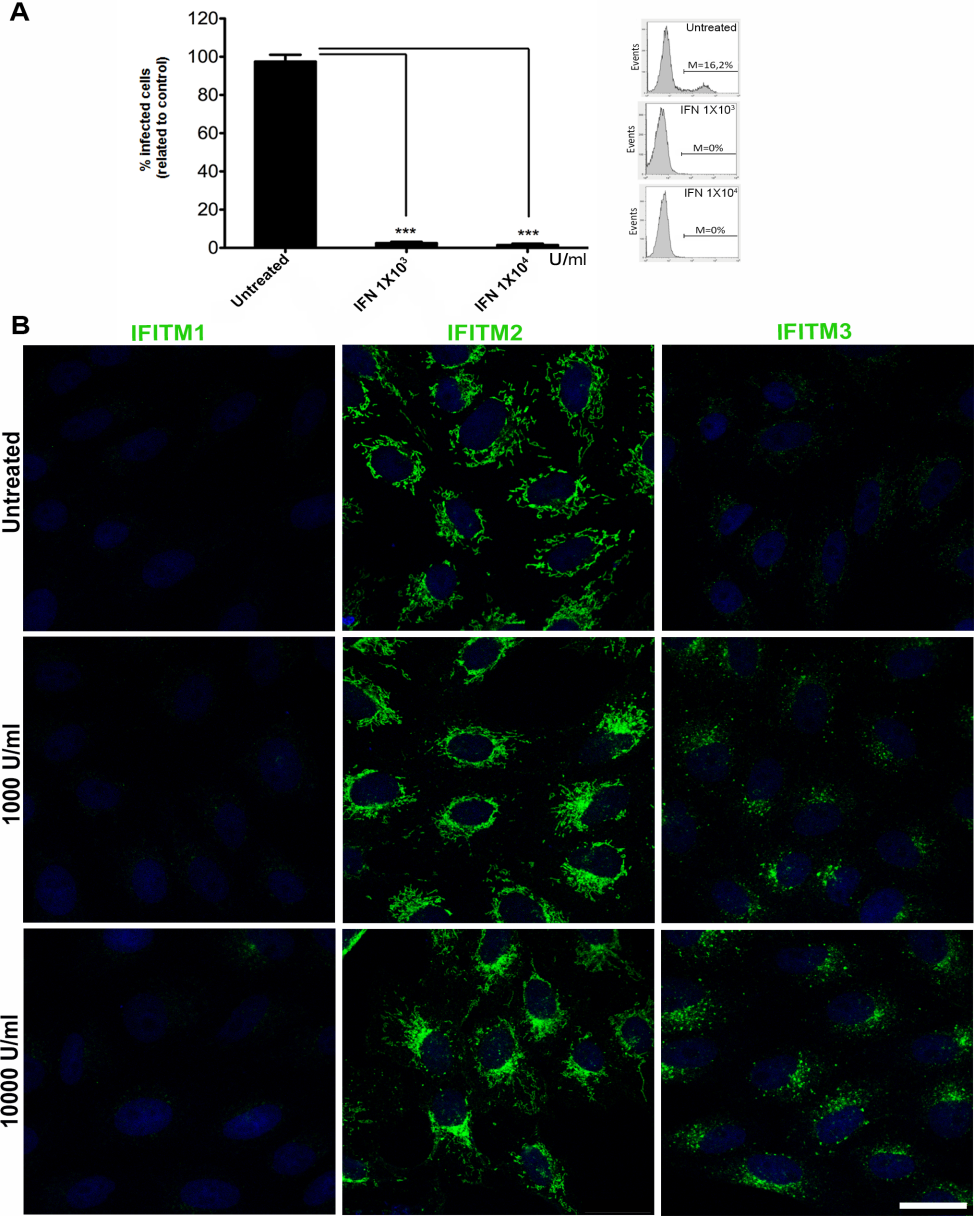
Effect of interferon and IFN-induced proteins IFITMs in ASFV infection. (A). ASFV-infected cells percentages analyzed by flow cytometry at 16 hpi in Vero cells untreated or treated with IFN at 1,000U/ml or 10,000U/ml 24 h prior to infection and infected with recombinant ASFV B54GFP at a MOI of 5 pfu/cell. Data are expressed as mean±SD of three independent experiments. Statistical significance was evaluated by one-way ANOVA followed by Bonferroni’s multiple comparison test. Differences are marked with asterisks as indicated (***p<0.001). An example of a typical FACS profile is shown. (B). Confocal fluorescence images of IFITM1, 2 and 3 subcellular distribution in untreated Vero cells or upon increasing universal IFN concentrations (1,000 or 10,000U/ml) for 24 h. Bar = 10μm.

To determine the effect of interferon on ASFV infection, Vero cells were pretreated with 1,000 or 10,000 U/ml of universal type-I IFN (PBL Assay Science) and 24 h later, they were infected with recombinant ASFV B54GFP at a MOI of 5 pfu/cell (Fig 1). Viral infection was quantified by analyzing the number of GFP-positive cells by flow cytometry at 16 hpi (Fig 1A). Pretreatment of Vero cells with universal type-I IFN at both concentrations completely abrogated ASFV infectivity when compared to untreated control cells. A sample flow cytometry profile is shown (Fig 1A).

### IFN treatment induces expression of IFITM proteins

IFITM proteins are located in different cellular compartments and their antiviral properties strongly correlate with their capacity to alter the fluidity and fusion ability of the membranes in which they reside [23, 24]. Then, we analyzed IFITMs expression and distribution in Vero cells upon IFN treatment. To this end, cells were incubated with either 1,000 or 10,000 U/ml of universal type-I IFN for 24 h and IFITMs 1, 2 and 3 distribution was analyzed by confocal microscopy (Fig 1B). Although basal levels of IFITM2 and 3 were detected prior to treatment with IFN, both proteins were clearly overexpressed after treatment and presented a characteristic vesicular distribution packed tightly to the nuclear area.

**Fig 2:**
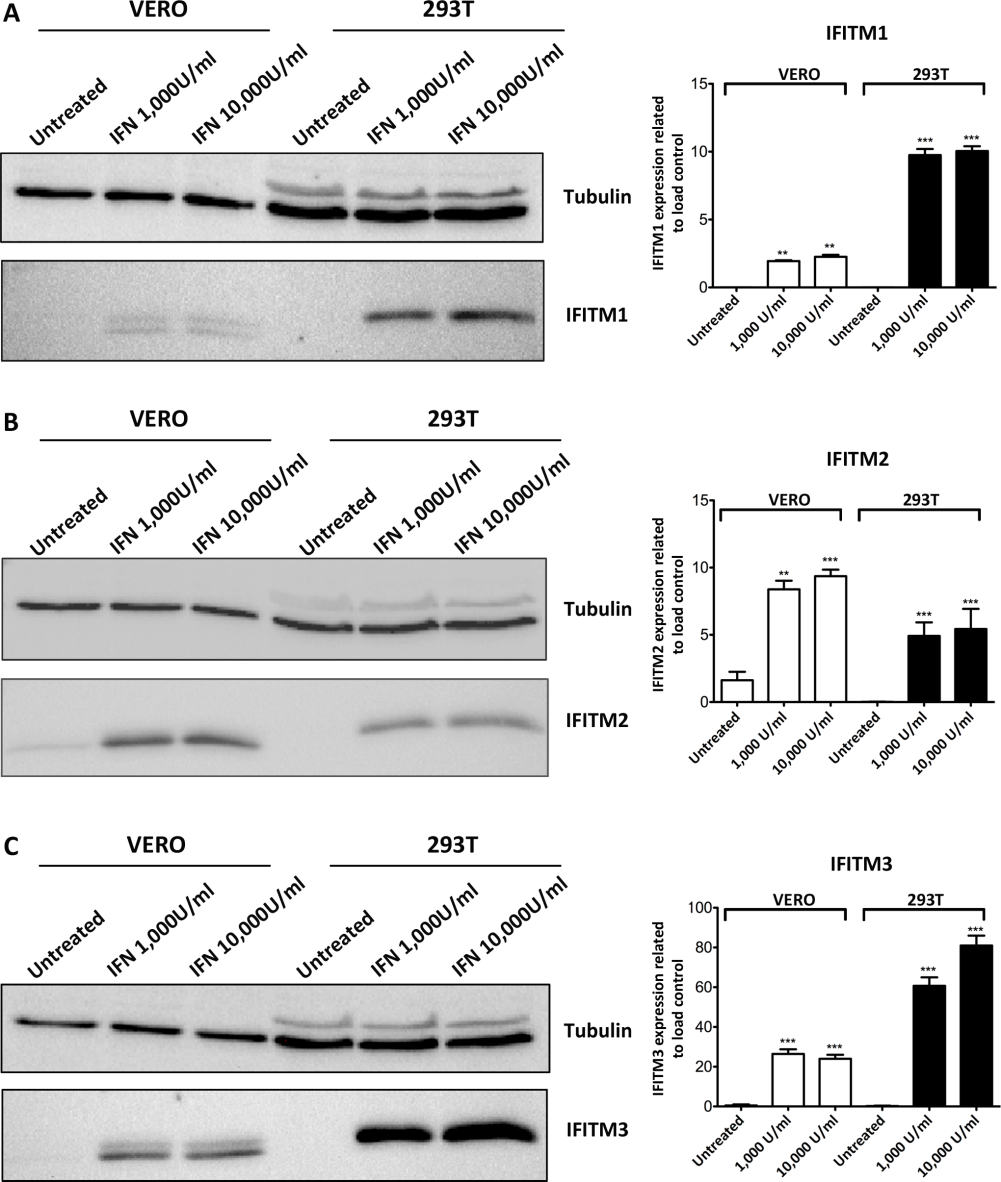
Expression of IFITM proteins upon interferon induction. Expression of IFITM proteins was induced in Vero and 293T cells after treatment with Universal type-I IFN at both 1,000 or 10,000 U/ml concentrations for 24 h and compared with untreated cells. Total cell extracts were incubated with antibodies against IFITM1 **(**A), IFITM2 (B) and IFITM3 (C). Right side panels represent WB quantification by densitometry for each IFITM and are expressed as the mean±SD from three independent experiments. Statistical significance was evaluated by a one-way ANOVA followed by Bonferroni’s multiple comparison test. Differences are marked with asterisks as indicated (**p<0.01; ***p<0.001).

To further analyze the expression of IFITM1, 2 and 3 upon IFN treatment, Vero and 293T cells were incubated with universal type-I IFN for 24 h and analyzed by WB (Fig 2). While IFITM1 and IFITM2 were induced by IFN in both cell lines (Fig 2A and 2B), IFITM3 showed the highest induction (Fig 2C).

### Generation and validation of IFITM-expressing cell lines

**Fig 3:**
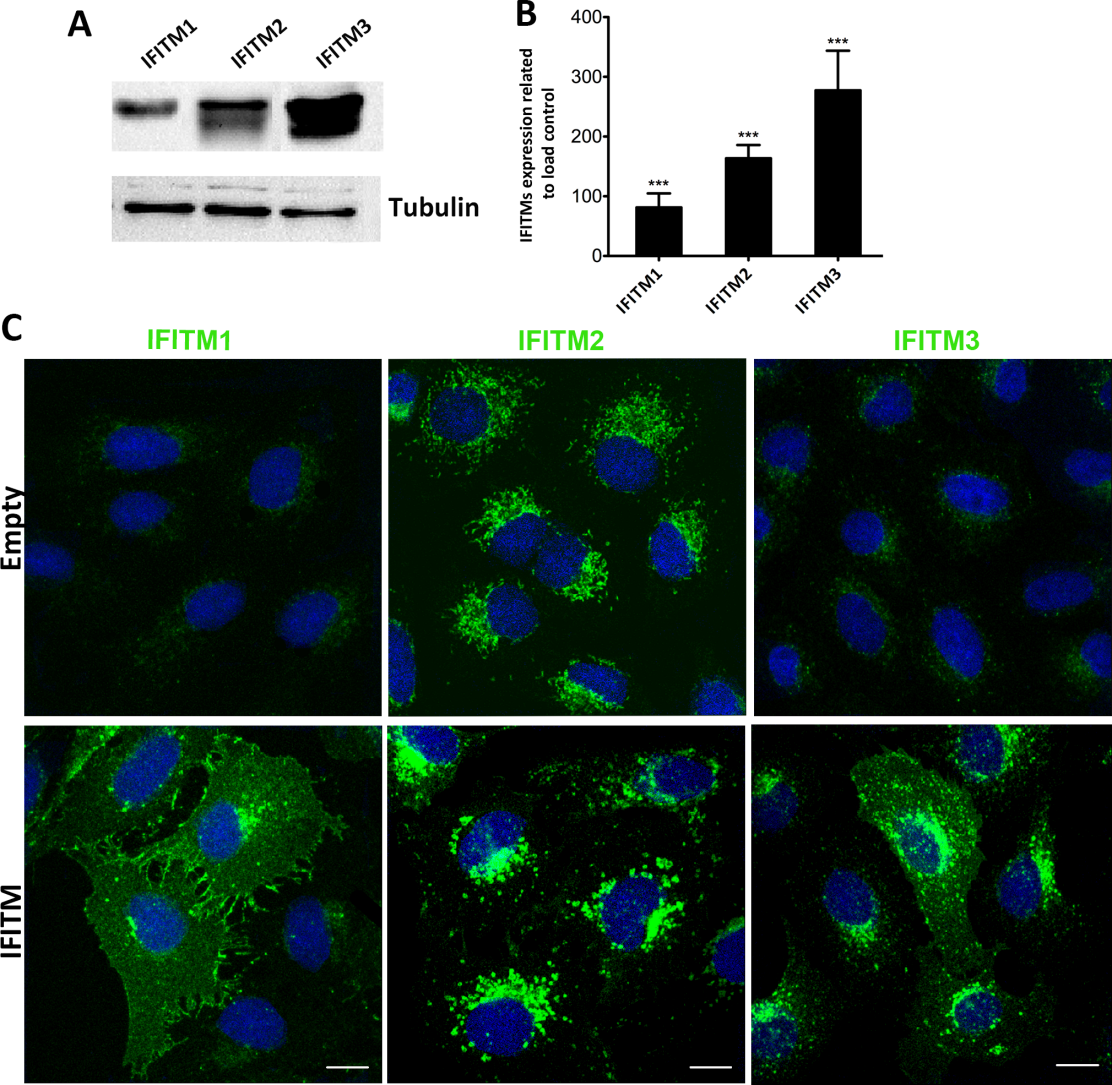
Validation of IFITM-expressing cell lines. (A). Western blot of Vero cell lines expressing IFITM1, 2 and 3. (B). Graphics depict mean±SD of the densitometry values relative to load control tubulin from three independent experiments. Statistical significance was evaluated by a one-way ANOVA followed by Bonferroni’s multiple comparison test. Differences are marked with asterisks as indicated (***p<0.001). (C). Subcellular distribution of IFITM in stable cell lines analyzed by confocal microscopy and compared to control cells containing the empty vector. Bar = 10μm.

In order to analyze the possible impact of IFITMs in ASFV infection, we generated Vero cells stably expressing the human IFITM1, 2, 3 (hereinafter referred to as Vero-IFITM1, 2 or 3 cells respectively) or control cells containing the empty vector. To generate the stable cell lines we used a lentiviral transduction system. Our proteins of interest were cloned into the pLVX vector (see material and methods section for detailed experimental procedures). Positively transduced Vero stable cells were selected with 8 μg/ml of puromycin. Once these cells were established, the expression of different IFITM proteins was confirmed by WB analysis (Fig 3A). As shown in the corresponding WB densitometry (Fig 3B), the highest expression levels within the IFITM family members corresponded to IFITM3, followed by IFITM2 and IFITM1.

After assessing the expression levels of the Vero-IFITM cells, we next wanted to ascertain the subcellular distribution of each IFITM. Expression of IFITM1, 2 and 3 in Vero-IFITM cells was compared with the distribution of IFITMs in Vero cells with the empty vector. Confocal microscopy experiments revealed that, IFITM1 was mainly distributed at the plasma membrane and to a lesser extent in perinuclear compartments, resembling endosomal structures (Fig 3C, lower left panel), while endogenous IFITM1 was barely detected in Vero cells containing the empty vector (Fig 3C, upper left panel).

In Vero-IFITM2 cells, overexpression led to a high accumulation of the protein in the perinuclear region, colocalizing with vesicular structures that resembled endosomes (Fig 3C, medium lower panel). Consistent with Fig 1B, there was a significant expression of endogenous IFITM2 in control Vero cells containing the empty vector, which displayed a mitochondria-like pattern together with vesicular-like structures.

Finally, overexpressed IFITM3 was found predominantly accumulated in the perinuclear region, showing a pattern consistent with localization at clustered endosomal structures (Fig 3C, lower right panel). Endogenous IFITM3 was barely detected in Vero cells containing the empty vector (Fig 3C, upper right panel).

**Fig 4:**
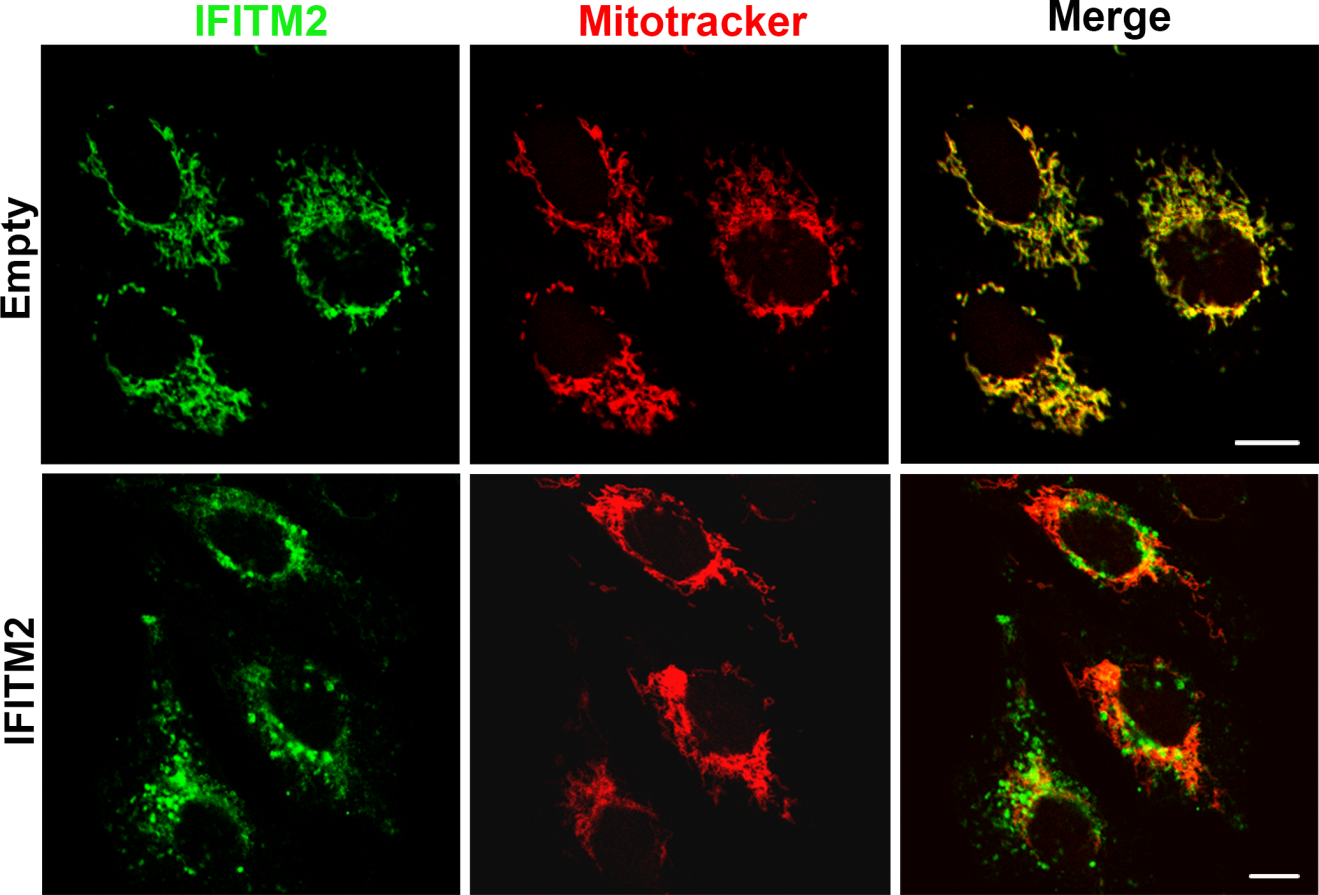
Differences in subcellular localization between endogenous and exogenous IFITM2. Expression of IFITM2 (green) was analyzed in Vero-IFITM2 cells and Vero cells containing the empty vector. Mitochondrial structures were marked with Mitotracker (red) to highlight their marked colocalization with IFITM2, especially in cells containing the empty vector. Bar = 10 μm.

Analysis of endogenous IFITM2 expression in control Vero cells suggested a mixed mitochondrial and vesicular pattern. To confirm this observation, we analyzed subcellular localization of IFITM2 using Mitotracker Red as a specific marker for mitochondria. Confocal images revealed a distribution of IFITM2 to mitochondrial structures in cells containing the empty vector (Fig 4). However, in Vero-IFITM2 cells, IFITM2 labelling localized to endosomal-like structures and we found few areas of colocalization between IFITM2 and mitochondria (Fig 1B).

### IFITM2 and IFITM3 alter distribution of endosomal compartments

**Fig 5:**
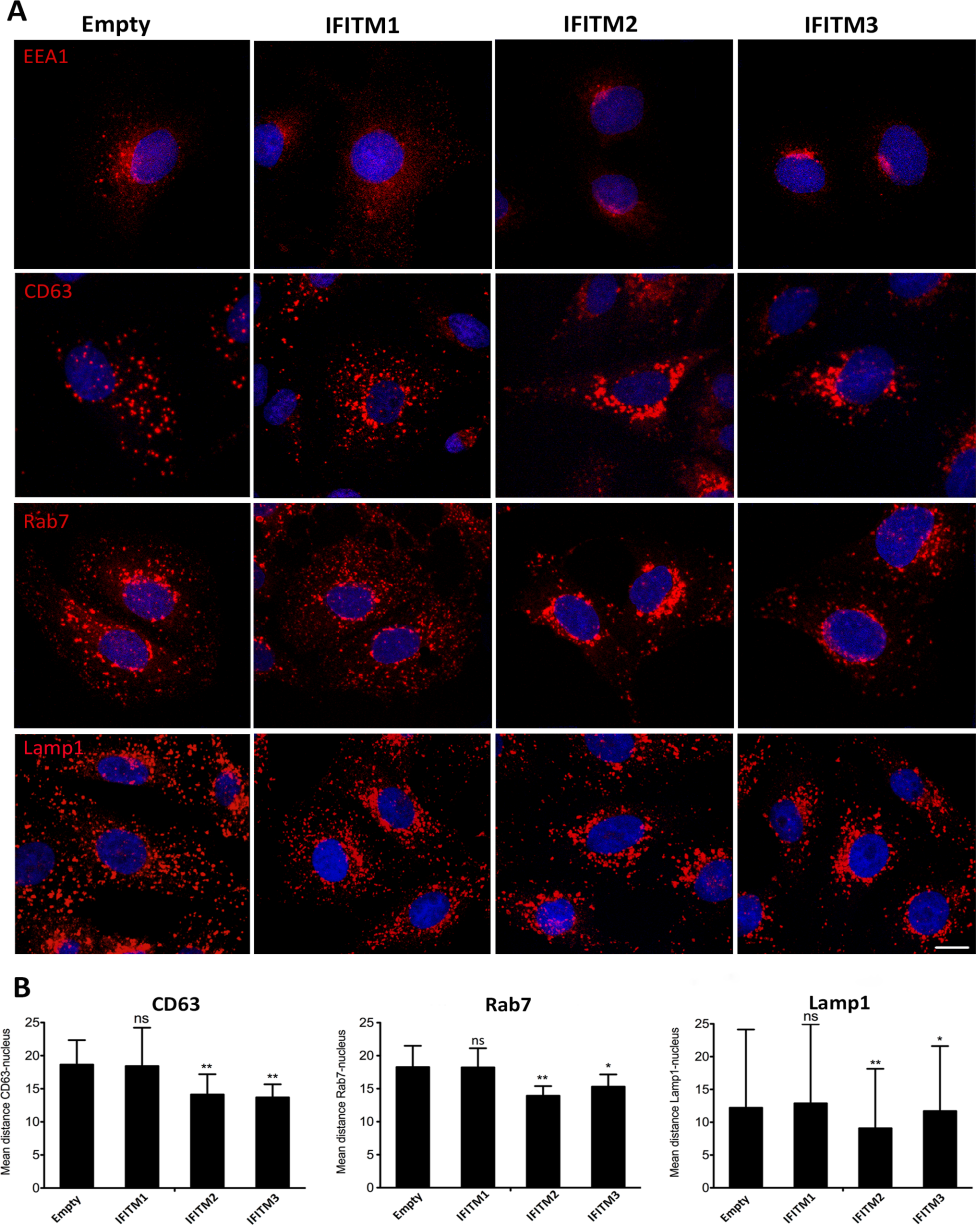
IFITM expression induces a redistribution of endosomal compartments. (A). Confocal microscopy images of Vero-IFITM cells or controls containing the empty vector stained with endosomal markers EEA1 (EE), CD63 (MVB), Rab7 (LE) and Lamp1 (LY). Endosomes were predominantly dispersed in the cytoplasm of cells containing the empty vector or concentrated to the perinuclear area in Vero-IFITM2 and 3 cells. (B). The change in distribution was quantified by measuring the mean distance to the nucleus of the different endosomal markers in x, y and z planes as described in materials and methods section. As shown in graphics, distance was reduced in Vero-IFITM2 and 3 cells. Graphics depict mean±SD of N = 30 cells per condition. Statistical significance was evaluated by a one-way ANOVA followed by Bonferroni’s multiple comparison test. Differences are marked with asterisks as indicated (*p<0.05; **p<0.01).

Then, we investigated the possible mechanism underlying the inhibition caused by IFITM in the viral infection. To further analyze the vesicular localization of IFITMs, we studied the expression of endosomal markers EEA1 (early endosomes; EE), CD63 (multivesicular bodies; MVB), Rab7 (late endosomes; LE) and Lamp1 (lysosomes; LY) in Vero-IFITM cells or control cells containing the empty vector (Fig 5A). Endosomes are normally distributed through the cytoplasm and this dispersed distribution was found for the different maturation stages of endosomes in controls and in Vero-IFITM1 cells. However, in Vero-IFITM2 and IFITM3 cells dispersed distribution changed and endosomes aggregated around the nucleus (Fig 5A). This endosome redistribution was analyzed by confocal microcopy in x, y, z planes by measuring the mean distance between each endosomal marker and the cell nucleus, using the “Distance Measure” ImageJ plug-in (Fig 5B). A total of 30 cells were analyzed for each condition.

We concluded that overexpression of IFITM2 and IFITM3 altered endosome distribution by accumulating these vesicles to the perinuclear region, similar to the pattern previously found after IFN treatment of Vero cells (Fig 1B) and this redistribution might reflect alterations in endo-lysosomal maturation and function.

### Colocalization of IFITMs with endosomal compartments

Next, we analyzed the colocalization rate between IFITMs and endosomal structures. CD63 remains mainly associated with intracellular vesicular membranes and it is particularly abundant in endosomal structures called multivesicular bodies (MVBs), which constitute a late and acidic endosomal compartment filled with intraluminal vesicles (ILVs). These ILVs are filled with cholesterol and are crucial for endosomal membrane backfusion and necessary for late endosome maturation.

**Fig 6:**
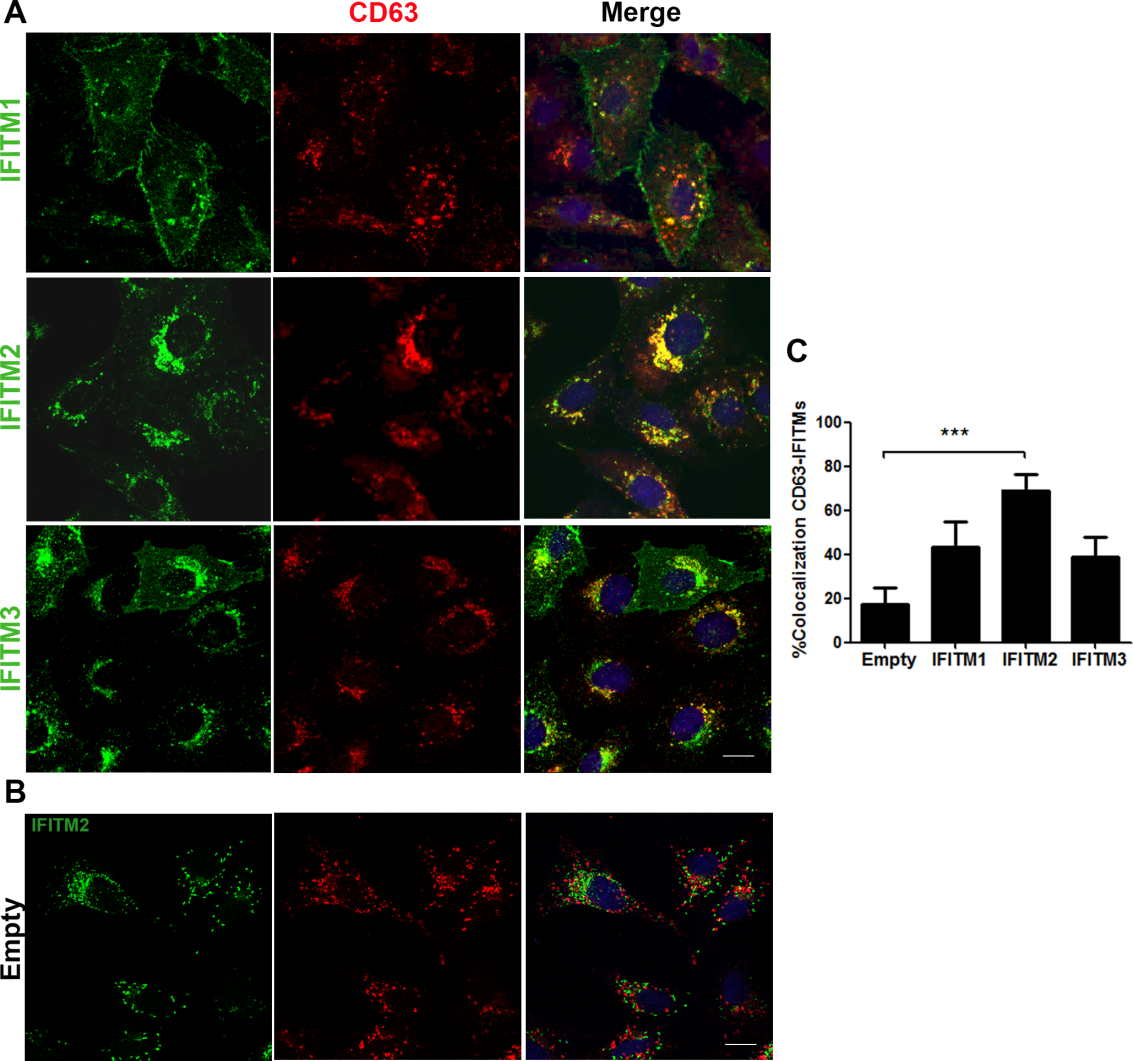
Colocalization of IFITMs with endosomal compartments. (A). Expression of IFITM1, 2 and 3 proteins (green) and CD63-positive late endosomes (red) in Vero-IFITM cells or cells containing the empty vector. (B). Distribution of IFITM2 (green) and CD63 (red) in cells containing the empty vector. Bar = 10μm. (C). Data were plotted on graphics representing the colocalization values between IFITM2 and endosomes in Vero-IFITM2 relative to control cells of N = 30 cells per condition. Statistical significance was evaluated by a one-way ANOVA followed by Bonferroni’s multiple comparison test. Differences are marked with asterisks as indicated (***p<0.001).

Co-staining of IFITMs and CD63 by immunofluorescence assay revealed a clear colocalization of IFITMs and CD63 in Vero-IFITM cells, with 75% colocalization for Vero-IFITM2 cells and 40% in Vero-IFITM1 and in IFITM3 cells (Fig 6A). Then, IFITM2, and to a lesser extent IFITM1 and 3, were located primarily in endosomal compartments as indicated by higher colocalization with endosomal marker CD63 (Fig 6C) when compared to the empty vector (Fig 6B).

### IFITM2 and IFITM3 induce accumulation of cholesterol in endosomal compartments

**Fig 7:**
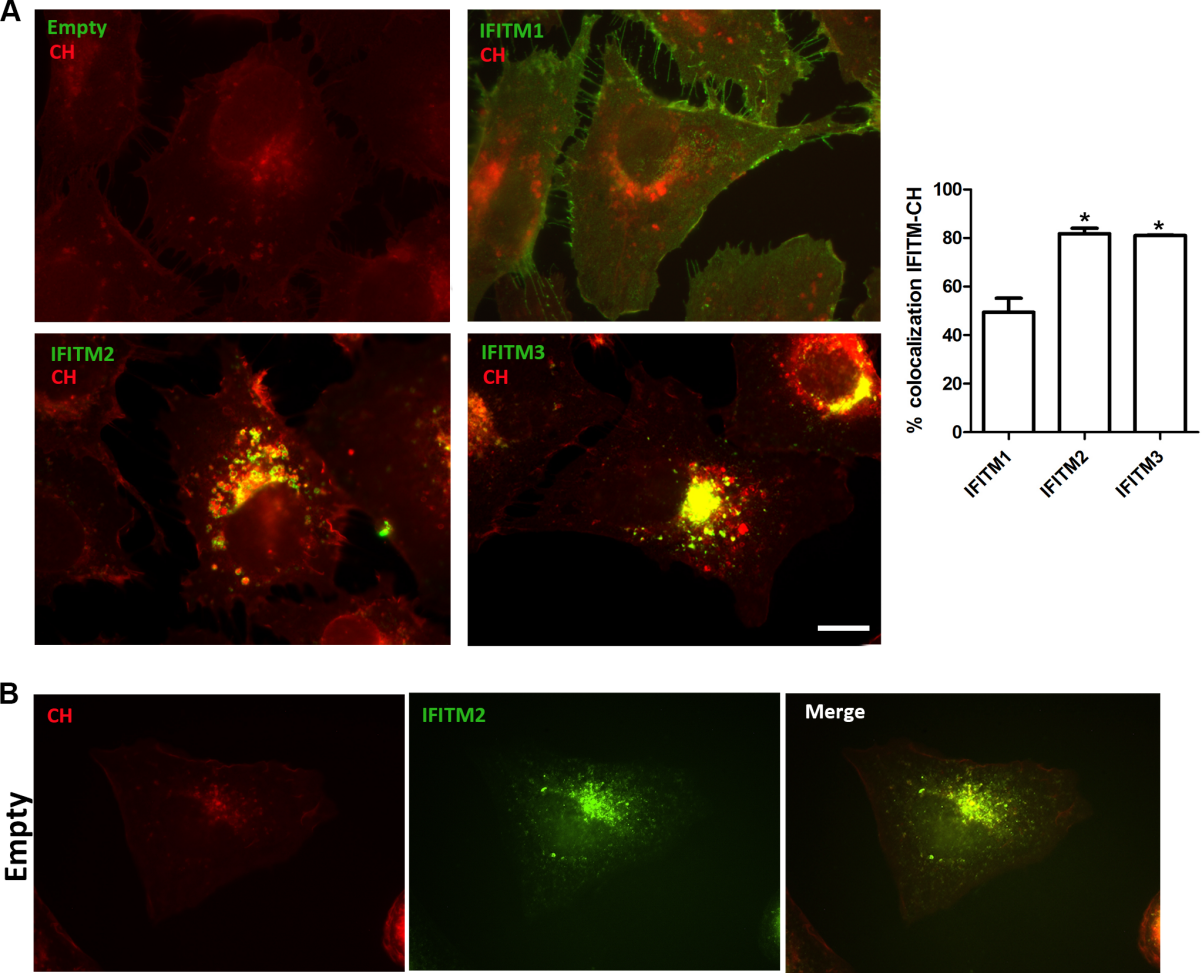
IFITM3 and 2 induce accumulation of cholesterol in endosomal compartments. (A). Analysis of cholesterol distribution using the specific marker filipin (red) in Vero-IFITM1, 2 and 3 cells (green) and cells containing the empty vector. Cholesterol is accumulated in endosomes upon expression of IFITM2 and 3. (B). Basal IFITM2 expression in control cells containing the empty vector did not produce cholesterol accumulation. N = 30 cells per condition. Bar = 10μm.

IFITM proteins could reduce curvature of cell membranes for fusion pore formation [24, 25] at the outer endosomal membrane, also called endosomal-limiting membrane, to differentiate it from the membranes of intraluminal vesicles inside multivesicular bodies (MVB/LE). This would lead to an alteration of membrane fusion and impaired cholesterol endosomal efflux. Changes in endosomal distribution such as those found in IFITM stably expressing cells (Fig 5) is a characteristic phenotype of an altered cholesterol endosomal efflux [23, 26]. Conversely, a conserved endosomal cholesterol efflux is required for a correct endosomal function. Therefore, we analyzed intracellular and intra-endosomal cholesterol levels by using the cholesterol marker filipin. Vero-IFITM2 and IFITM3 cells showed intense intracellular cholesterol accumulation at the perinuclear area (Fig 7A) that was absent in control cells containing the empty vector (Fig 7B). This cholesterol accumulation also colocalized with the IFITM-labeled endosomal vesicles. In contrast, in Vero-IFITM1 cells, only discrete colocalization areas between IFITM1 and cholesterol were found at the plasma membrane.

Collectively, these findings indicate that IFITM2 and IFITM3 overexpression in Vero cells results in cholesterol accumulation in endosomal compartments, and as a result it might be responsible of an altered endosomal function possibly altering viral entry through this pathway.

### IFITM2 and IFITM3 proteins restrict ASFV entry

Next, we investigated whether overexpression of IFITMs could restrict ASFV infection or not. ASFV is known to require acidic endosomal compartments for entry into host cells. Successful ASFV entry and egress from endosomes depends on the acidic pH of late endosomes [11]. Previous experiments in the laboratory revealed that the inhibition of acidification impaired viral decapsidation and viral particles were retained in Rab7-positive late endosomes, thus blocking viral infection progression [11]. Given that IFITM restriction could be mediated at the endocytic pathway [1, 4], we hypothesized that IFITM overexpression may affect virus entry process and subsequent ASFV infection.

Acidic pH of late endosomal compartments is required for viral decapsidation, which is the first step required for uncoating previous to endosomal escape of the virus and the start of replication [11]. The ASF virion is composed of several concentric domains. The viral capsid is formed by major capsid protein p72 organized in capsomers. Hence, after decapsidation, p72 staining of virions is lost. The internal core is composed by the nucleoid containing genome coated by the core shell, a thick protein layer that can be labeled with antibodies against p150, a core shell protein derived from processed core shell polypeptides [27].

**Fig 8:**
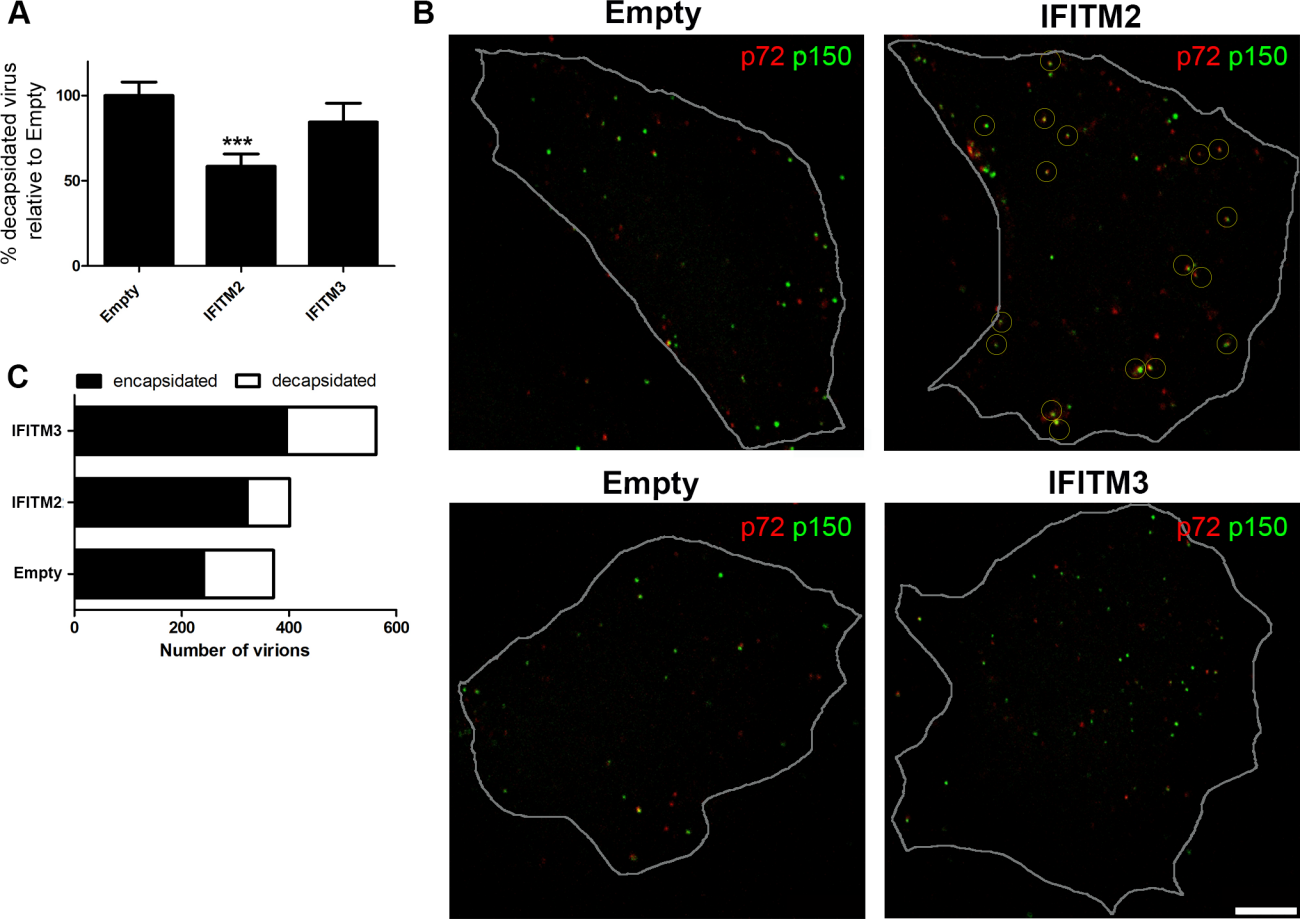
IFITM2 and IFITM3 restrict ASFV entry. (A). Analysis of the decapsidated virions in Vero-IFITM2 and IFITM3 compared to Vero control cells containing the empty vector. IFITM2 significantly decreased numbers of decapsidated virions. Graphics show the percentage of decapsidated virions relative to controls. Statistical significance was evaluated by a one-way ANOVA followed by Bonferroni’s multiple comparison test. Differences are marked with asterisks as indicated (***p<0.001). (B). Confocal microscopy images of ASFV virions labelled for viral major capsid protein p72 (red) and inner core protein p150 (green) upon expression of IFITM2, IFITM3 or empty vector. Positive virions for both proteins yielded a yellow signal and are shown encircled. Bar = 10μm. (C). Graphical representation showing the ratio of decapsidated and encapsidated virions in Vero-IFITM2, IFITM3 or empty control cells related to the total number of virions counted from a total of N = 15 cells per condition.

We used antibodies against the major viral capsid protein p72 to detect viral capsids and against viral core protein p150 to detect viral cores (Fig 8). We analyzed the number of encapsidated viral particles, double labeled for p72 and p150, and compared with the number of successfully decapsidated viral cores positive for p150 at 75 minutes postinfection (mpi). At this time point, most virions undergo uncoating and progress to replication in normal conditions, otherwise, encapsidated virions would accumulate. Confocal microscopy revealed that the number of viral cores was severely decreased in Vero-IFITM2 cells when compared to control cells containing the empty vector (Fig 8B). Double-labeled encapsidated viral particles were counted and the ratio of decapsidated viral cores compared to the total number of virions per individual cell was calculated and expressed in percentages (Fig 8A). The percentage of decapsidated virus severely decreased under IFITM2 expression. However, IFITM3 expression produced an accumulation of virions leading to higher numbers of total virions (Fig 8C). This increase in the number of total virions might be the result of an impaired progression of the ASFV replication cycle. These virions would neither proceed with infection nor be degraded and this would be consistent with an inhibition of the membrane fusion capacity at the endosomal level. Altogether, these results indicate that viral entry was the rate-limiting step in Vero-IFITM2 and IFITM3 cells for ASFV infectivity.

### IFITMs impair ASFV infectivity

**Fig 9:**
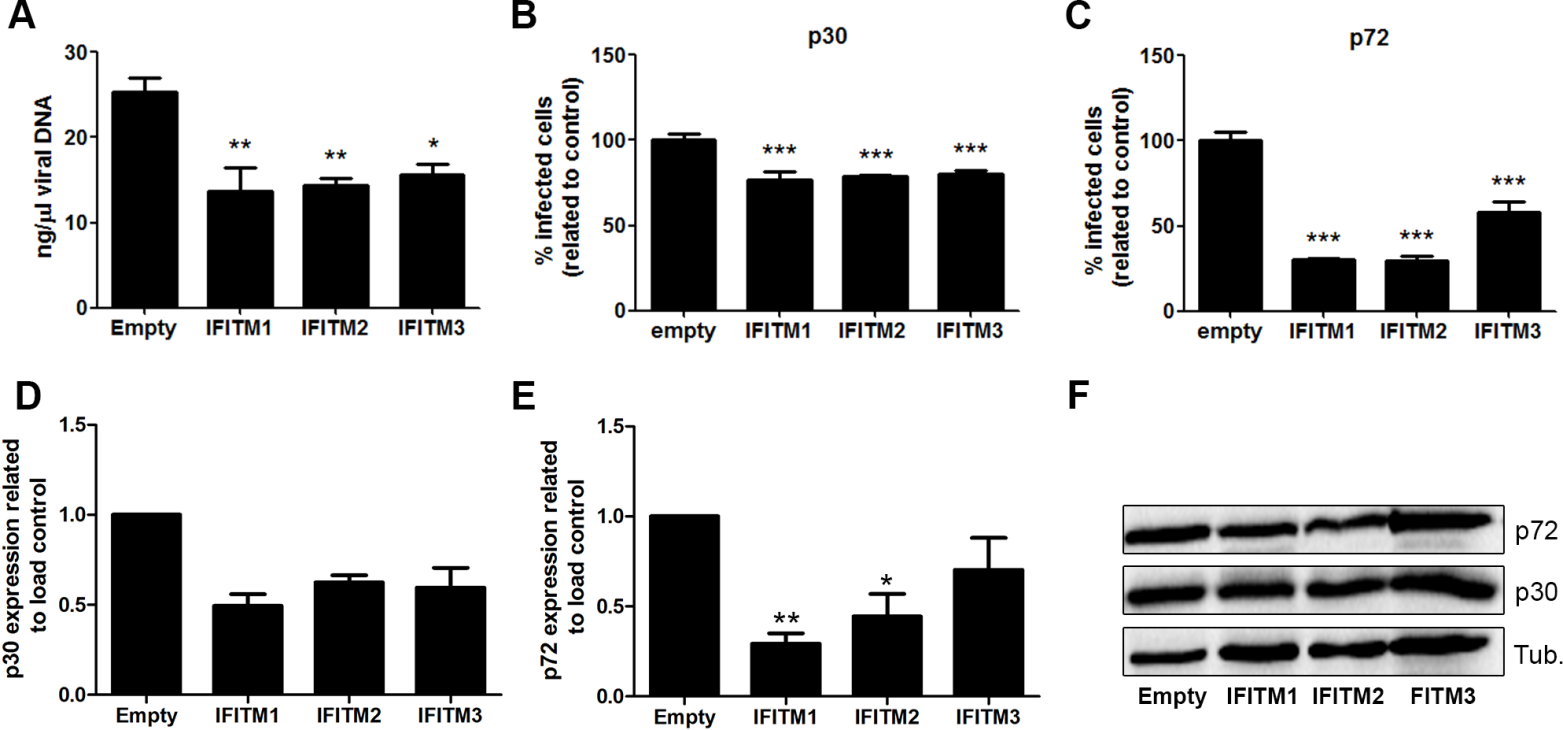
General impact of IFITM overexpression in several infection parameters. (A). Quantitation of ASFV viral DNA at 16 hpi in Vero-IFITM cells compared to empty controls. (B-C). Flow cytometry analysis showing percentages of infected Vero-IFITM cells at a MOI of 1 pfu/cell relative to empty controls evaluated by early p30 expression at 6 hpi (B) or late p72 protein expression at 16 hpi. (D-E). Viral protein expression in IFITM expressing cells. Vero-IFITMs and empty control cells were infected at a MOI of 1 pfu/cell. WB quantification of early p30 expression at 6 hpi (D) and late p72 expression at 16 hpi (E). (F). Western blot images of previous panels D and E. (A-E). Graphics depict mean±SD of densitometry values from three independent experiments. Statistically significance was evaluated by a one-way ANOVA followed by Bonferroni’s multiple comparison test. Differences are marked with asterisks as indicated (*p<0.05; **p<0.01; ***p<0.001).

Finally, we analyzed the impact of IFITM expression on other infection parameters to find reduced number of copies of ASFV genome at 16 hpi (Fig 9A). IFITM overexpression induced a consistent reduction in infectivity as measured by early protein p30 expression by flow cytometry (Fig 9B). However, these reductions were even more marked when analyzing infected cell percentages by late p72 protein expression (Fig 9C). Finally, we also analyzed viral protein expression by WB (Fig 9D–9F). The expression of protein p30 at 6 hpi (Fig 9D) and p72 at 16 hpi (Fig 9E) resulted significantly reduced as other infection parameters.

## Discussion

In the present work, we have studied the antiviral effect of the IFITM family of proteins in the context of cell-adapted ASFV infection in Vero cells. While different IFITM proteins have been repeatedly described as inhibitors of a broad spectrum of RNA viruses [6], their antiviral role involving DNA viruses is poorly studied and only reported in the Rana grylio virus (RGV), blocking the virions at the entry stage into the host cell [8].

ASFV Ba71V infection in Vero cells is significantly affected upon IFN treatment [28, 29]. In general, the sensitivity towards the induction of the innate immune response of the host cell has led viruses to acquire different strategies to regulate the IFN pathway for its own benefit. Such is the case of ASFV viral gene A276R, which negatively regulates the induction of IFN through targeting IRF3 in a NFκB-independent manner [30]. Another example is the ASFV gene I329L, which codifies for a viral TLR3 homolog with inhibitory activity against IFN [31]. Finally, the myxovirus resistance gene A (MxA), which is another Interferon Stimulated Gene (ISG), also inhibits the replication and the late gene expression of ASFV [32].

We report here that upon IFN treatment of Vero cells, the distribution of IFITM proteins changes into a perinuclear vesicular pattern resembling endosomes. Our analysis of endogenous IFITM2 expression in the absence of IFN induction showed colocalization with mitochondrial structures. Interestingly, IFITM2 underwent vesicular pattern redistribution around the cell nucleus when overexpressed and upon IFN treatment as well.

Our characterization of the cellular distribution of IFITM1, 2 and 3 also unveiled specific localization patterns linked to the endosomal pathway upon overexpression, particularly in the late endosomal compartments. This distribution is coincident with previously reported data of other groups, which described localization of endogenous IFITM1 at the plasma membrane and early endosomes, and of IFITM2 and IFITM3 in late endosomes and lysosomes [33, 34]. Interestingly, overexpression of IFITM1 has been recently described to delay the proteolytic degradation of human papilloma virus (HPV) capsids in keratinocytes [9]. This, however, did not affect the replication of the virus.

Our analysis of ASFV Ba71V uncoating correlated the expression of IFITM2 with a decrease in the numbers of decapsidated ASF virions in Vero cells. This suggests a possible role for IFITM2 in inhibiting ASFV exit from late endosomes. In contrast, IFITM3 did not modify the ratio between encapsidated and decapsidated virions. Instead, IFITM3 apparently increased the accumulation of virions that are probably retained and do not proceed to degradation and/or to a productive infection. These results may also suggest that the presence of IFITM3 affects the release of the virions from the late endosomal compartments.

ASFV enters into the host cell by dynamin-dependent and clathrin-mediated endocytosis [12, 17] and macropinocytosis [35]. In fact, endosomal pathway integrity is known to be important for ASFV infection, both for culture-adapted isolates in cell lines [11] or for virulent and attenuated isolates in primary macrophages [12]. Hence, it is not surprising that infection could be impaired by these restriction factors acting at the endosomal membrane.

The aforementioned IFITM2- and IFITM3-mediated inhibition of ASFV entry has been previously reported in other viruses, including IAV, flaviviruses (DENV and WNV) [6], filoviruses (MARV and EBOV) and coronaviruses (such as SARS) [34]. In contrast, infection with alphaviruses, arenaviruses and MLV (a retrovirus) seems to be unaffected by IFITM protein expression [4]. In general, IFITM-mediated viral inhibition has been related to impaired viral-host membrane fusion subsequent to viral binding and endocytosis [33, 34]. IFITM3 has also been reported to modulate the fluidity and the bending modulus of the cell membrane, thus making it resistant to viral fusion machinery [36].

We also studied whether the inhibition of ASFV entry could be due to an alteration of the endosomal compartments. Analysis of endosome distribution upon IFITM overexpression revealed that IFITM2 and IFITM3 altered the normal distribution of early and late endosomes and lysosomes. However, this alteration was not found in the presence of IFITM1. A collapse of endosomes to the perinuclear area is also a characteristic phenotype of an alteration of endosomal cholesterol efflux [26]. Also, recent publications from our laboratory demonstrated that accumulation of cholesterol in endosomes caused by a chemical inhibition of cholesterol flux, causes virion retention inside endosomes and inhibition of infection progression [37].

There are currently two proposed models trying to explain the IFITM-mediated inhibition of viral entry. The first one, known as the “tough membrane” model, postulates that intramembranous interactions between adjacent IFITMs alter the fluidity and bending of the host cellular membrane, making it resistant to the viral fusion machinery [36]. The second model suggests that IFITMs can induce the accumulation of cholesterol in the endosomal membrane and membrane fusion defects [38] disturbing intracellular cholesterol homeostasis that finally blocks the viral release into cytosol [23].

IFITM2 and IFITM3 have been previously reported to alter the cholesterol homeostasis at the late endosome, leading to cholesterol accumulation and blocking the viral release [23]. In the present study, we have described accumulation of cholesterol upon overexpression of IFITM2 and IFITM3. We have recently reported that inhibition of cholesterol exit from endosomes using chemical inhibitors causes retention of virions inside these vesicles, thus impairing progression of the infection [37]. Altogether these data could suggest that the antiviral action of IFITMs may affect to a higher extent to those viruses that require the endosomal pathway during the early stages of infection.

Collectively, our results illustrate a close relationship between the IFITM protein family and the endosomal pathway, leading to the inhibition of ASFV infection. The antiviral action of IFITMs could rely on alterations of the endosomal physiology and ongoing studies in our laboratory will be focused on antiviral targets at the molecular regulation of late endosomes. Also, a role in cell-to-cell viral transmission has been postulated for IFITMs, which are incorporated into HIV-1 virions [39].

We have performed this study in a cellular system using the cell-adapted Ba71V isolate in Vero cells but these results will be next extended to other ASFV isolates with porcine macrophages. Further studies will be required for better understanding the relevance of IFITMs in the context of ASFV infection. In summary, IFITMs represent a broad and previously unappreciated class of restriction factors that degrade invading enveloped viruses and may therefore be considered as potential antiviral components to protect the host cell.
